# Mesalamine Associated Bradycardia

**DOI:** 10.7759/cureus.2425

**Published:** 2018-04-04

**Authors:** Michael Krzyzak, Anupam Gupta, Evgeny Antonov, Stephen M Mulrooney

**Affiliations:** 1 Department of Medicine, Staten Island University Hospital, Northwell Health, Staten Island, USA; 2 Department of Nursing, Staten Island University Hospital, Northwell Health, Staten Island, USA; 3 Department of Gastroenterology, Staten Island University Hospital, Northwell Health, Staten Island, USA

**Keywords:** ulcerative colitis, bradycardia, drug side effects

## Abstract

A 38-year-old female presented with an acute flare of ulcerative colitis. She was started on prednisone and mesalamine. Within 24 hours of initiating mesalamine, she developed sinus bradycardia. After holding mesalamine, the heart rate returned to normal within five days. Our case illustrates the third described case, to our knowledge, of severe sinus bradycardia secondary to mesalamine.

## Introduction

Since the 1960s, mesalamine was used to treat ulcerative colitis [1]. Mesalamine has shown safety and efficacy in treating ulcerative colitis [2]. Here we describe the third known case of mesalamine inducing bradycardia [3].

## Case presentation

A 38-year-old female presented to the hospital with complaints of lower abdominal pain and diarrhea with rectal bleeding for two weeks. She had a history of iron deficiency anemia and ulcerative colitis. Family history was significant for hypertension in both parents. She was diagnosed with ulcerative colitis six years prior with colonoscopy and biopsy. She tried numerous regimens of sulfasalazine and mesalamine with varying levels of remission, never requiring steroids. She last took mesalamine five years prior, with full remission. She did not report side effects to prior mesalamine regimens. At presentation, she was not on any home medication.

A computed tomography (CT) scan showed circumferential wall thickening and mucosal hyperenhancement of the sigmoid colon and rectum with enlarged subcentimeter pericolonic lymph nodes consistent with proctocolitis. Blood cultures, urine cultures, and fecal cultures were drawn and later found to have no growth. Stool for Clostridium difficile enteric toxin was negative. Hemoglobin was 7.3g/dL. Initial management began with intravenous fluid support, methylprednisolone, ciprofloxacin, and metronidazole. Once the abdominal pain began subsiding, a liquid diet and oral mesalamine were started. Within 24 hours of initiating mesalamine, she complained of dizziness and chest pain. An electrocardiogram performed showed sinus bradycardia at 37 bpm. On presentation to the hospital, however, she had a heart rate of 81 bpm.

**Figure 1:**
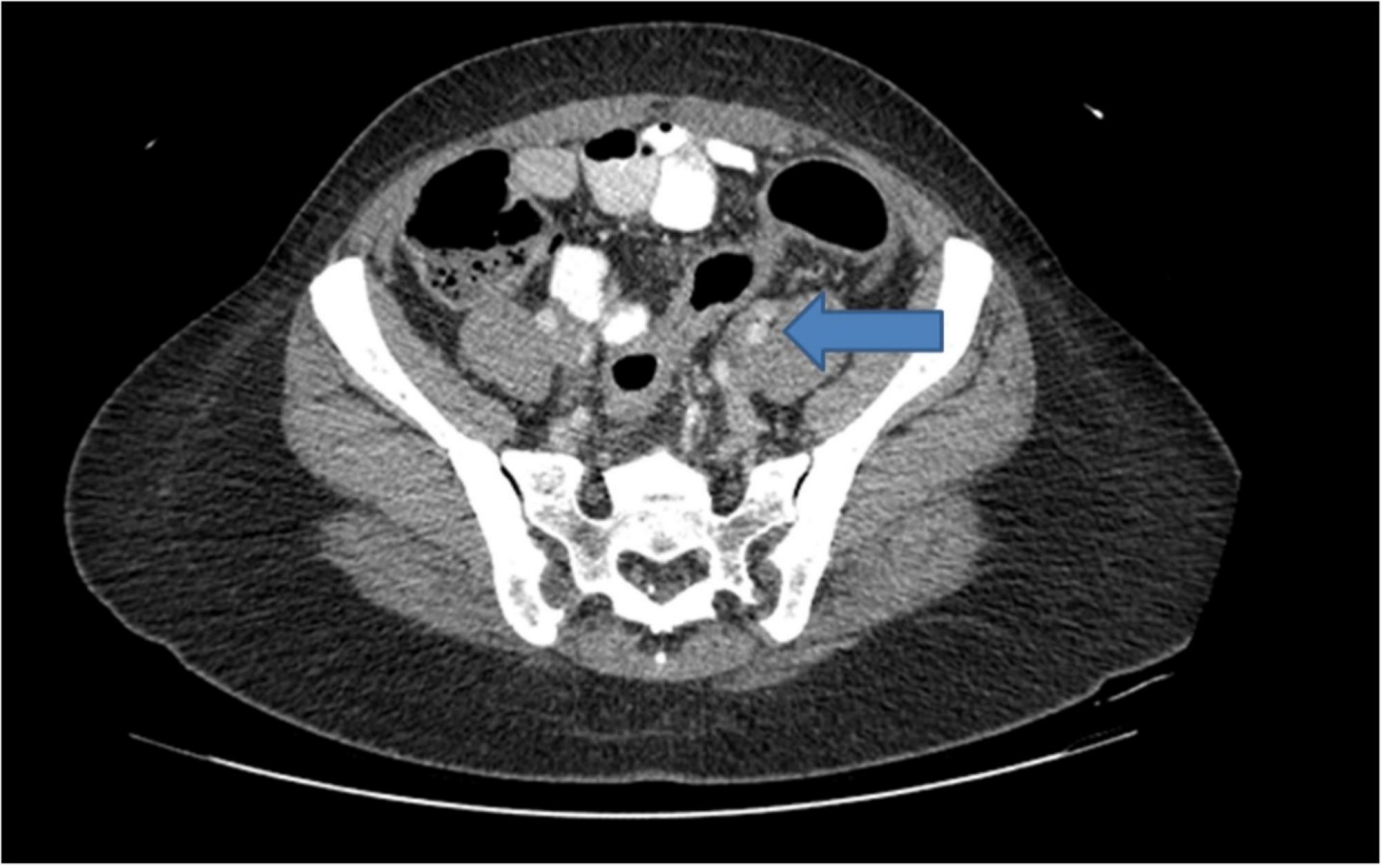
Computed Tomography of the Abdomen Circumferential wall thickening and mucosal hyperenhancement of the sigmoid colon and the rectum with surrounding inflammatory changes and several subcentimeter pericolonic lymph nodes (blue arrow).

Mesalamine was held. A cardiac workup was performed consisting of telemetry monitoring, cardiac enzymes, and echocardiogram. Cardiac workup was negative. For four days thereafter, the heart rate was persistently bradycardic with rates between 40-50 bpm.

**Figure 2:**
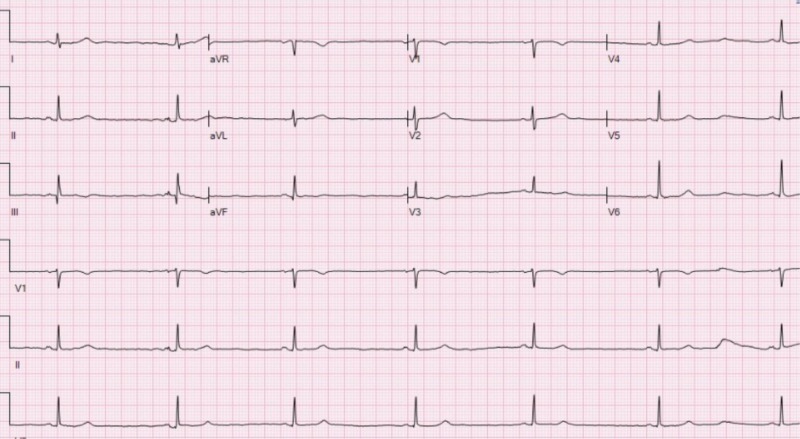
Electrocardiogram Showing Sinus Bradycardia

By day five, the heart rate returned to a range of 50 to 60 beats per minute. 

To treat the ulcerative colitis flare, intravenous methylprednisolone was changed to oral prednisone. Biologic agents were to be considered once the acute flare subsided. She was discharged home to follow up with gastroenterology for further workup and maintenance medication.

## Discussion

Sulfasalazine was first used to treat rheumatoid arthritis in 1942. It was applied to ulcerative colitis in the 1960s [1]. The active component is 5-aminosalicylic acid, or 5-ASA, along with sulfapyridine [4]. Mesalamine has a half life of approximately 25 hours (Delzicol (R) oral delayed-release capsules, mesalamine oral delayed-release capsules).

Side effects reported include intolerance, nausea, vomiting, skin eruptions, pancreatitis, hepatotoxicity, eosinophilic pneumonitis, and arthralgia. It is reported that 45% of patients receiving 5-ASA compounds experience an adverse reaction to some degree [5]. Its effects on the molecular level include inhibition of the activity of the nuclear factor-kappa B (NF-κB) pathway, inhibition of intestinal epithelial cell injury, inhibition of chemoattractant leukotrienes, and modulation of prostaglandin metabolism [6]. Prostaglandin administration has been shown to increase heart rate in experimental models [7-8]. Modulating the effect of prostaglandins may be contributory to the pathomechanism of bradycardia, as reported in our case report.

Sinus bradycardia has been reported earlier with mesalamine use [3,9]. Since the patient has had mesalamine in the past, a predisposing factor must be present which promotes bradycardia. The patient was not on any home medication and no new medications were started during this hospitalization.

## Conclusions

Our case is the third described case of mesalamine associated bradycardia. The patient was not rechallenged with mesalamine, and thus cannot fully prove causality. Although literature shows evidence that prostaglandin may play a role in altering heart rate, further research needs to be performed in order to understand the mechanism of action on the cardiovascular system as well as the side effects of mesalamine. Also, further research needs to clarify if there are susceptibilities to adverse conduction related abnormalities as a result of exposure to this class of medications.
